# Improved Performance of SRAM-Based True Random Number Generator by Leveraging Irradiation Exposure

**DOI:** 10.3390/s20216132

**Published:** 2020-10-28

**Authors:** Xu Zhang, Chunsheng Jiang, Gang Dai, Le Zhong, Wen Fang, Ke Gu, Guoping Xiao, Shangqing Ren, Xin Liu, Sanyong Zou

**Affiliations:** 1 Institute of Electronic Engineering, China Academy of Engineering Physics, Mianyang 621999, China; zhangxu@mtrc.ac.cn (X.Z.); jiangchunsheng@mtrc.ac.cn (C.J.); zhongle@mtrc.ac.cn (L.Z.); fangwen@mtrc.ac.cn (W.F.); guke@mtrc.ac.cn (K.G.); xiaoguoping@mtrc.ac.cn (G.X.); rensahngqing@mtrc.ac.cn (S.R.); liuxin@mtrc.ac.cn (X.L.); zousanyong@mtrc.ac.cn (S.Z.); 2 Microsystem and Terahertz Research Center, China Academy of Engineering Physics, Chengdu 610200, China

**Keywords:** SRAM-based TRNG, total ionizing irradiation (TID), min-entropy, data remanence effect, throughput

## Abstract

Encryption is an important step for secure data transmission, and a true random number generator (TRNG) is a key building block in many encryption algorithms. Static random-access memory (SRAM) chips can be easily available sources of true random numbers, benefiting from noisy SRAM cells whose start-up values flip between different power-on cycles. Embarking from this phenomenon, a novel performance (i.e., randomness and throughput) improvement method of SRAM-based TRNG is proposed, and its implementation can be divided into two phases: irradiation exposure and hardware postprocessing. As the randomness of original SRAM power-on values is fairly low, ionization irradiation is utilized to enhance its randomness, and the min-entropy can increase from about 0.03 to above 0.7 in the total ionizing irradiation (TID) experiments. Additionally, while the data remanence effect hampers obtaining random bitstreams with high speed, the ionization irradiation can also weaken this impact and improve the throughput of TRNG. In the hardware postprocessing stage, Secure Hash Algorithm 256 (SHA-256) is implemented on a Field Programmable Gate Array (FPGA) with clock frequency of 200 MHz. It can generate National Institute of Standards and Technology (NIST) SP 800-22 compatible true random bitstreams with throughput of 178 Mbps utilizing SRAM chip with 1 Mbit memory capacity. Furthermore, according to different application scenarios, the throughput can be widely scalable by adjusting clock frequency and SRAM memory capacity, which makes the novel TRNG design applicable for various Internet of Things (IOT) devices.

## 1. Introduction

To keep the communication between Internet of Things (IOT) devices secure, the data often require encryption before being transmitted [1], and true random number generators (TRNGs) are indispensable primary components in many encryption algorithms [2,3]. TRNGs usually extract random numbers from the outcome of non-deterministic physical processes, such as thermal noise of resistors [4], random jitter of clock signals [5], random telegraph noise (RTN) [6] and metastable oscillation of latch circuits [7]. Various TRNGs proposed in the literature can be further categorized into analog and digital ones according to the types of circuits. The analog TRNGs generally amplify thermal noise directly to a measurable level and then utilize a comparator to quantize it [8]. However, these analog schemes require elaborative calibrations to remove bias in generated random bits. Extensive analog designs also bring out difficulties in system integration [8]. Digital TRNGs have advantages over analog ones in terms of integration and robustness to environmental variations [8]. Researchers have proposed different digital TRNGs based on oscillator jitter [9], metastability [10] and other device noise. It is easy to design and verify ring oscillator jitter based TRNGs on FPGA, but their randomness is relatively poor [11]. Metastability-based solutions possess high throughput and power efficiency but often need run-time controlling logic to remove mismatches in devices [10].

There also exists one common drawback of these TRNG designs in that they cannot reuse standard integrated circuit components and are usually used as single-purpose circuits, which increases the cost of production and decreases usage flexibility [12]. Especially with advancement of ubiquitous IOT applications (e.g., smart home, wireless sensor networks, and even smart city), the secure data transmission become more and more of a concern [13]. However, these IOT devices are usually source-constrained and lack dedicated hardware security components such as TRNGs, which leads to poorly encrypted communication, or often no encryption at all. Thus, it will be attractive to reuse existing standard components as primary security components, and static random-access memory (SRAM) is a potential option. It has been reported that memories will consume over 70% of active area, most being SRAM [13]. SRAM is available in many digital integrated circuits and system-on-chips [14], and thus there is no need for additional hardware. It is easy and low-cost to integrate commercial SRAM chips into IOT devices and reuse them as TRNGs [15,16,17].

**Figure 1:**
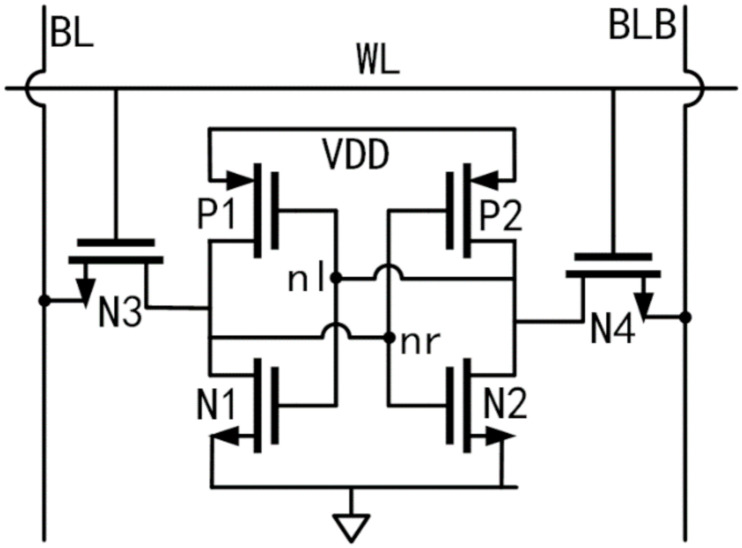
Schematic of a six-transistor (6-T) static random-access memory (SRAM) cell.

The source of randomness of SRAM-based TRNGs is the noisy power-on values of SRAM cells [15]. One SRAM chip contains large amounts of six-transistor (6-T) cells, as displayed in Figure 1. When it is powered up, each cell in the chip is randomly initialized to ‘0′ or ‘1′ depending on the random mismatches deduced by production variations [18]. Then, a binary power-on sequence will be generated; this sequence can be used to derive a chip fingerprint or generate a true random number hinging on its entropy [15,19]. In experimental evaluations, the min-entropy is usually regarded as the worst-case (i.e., the greatest lower bound) measure of randomness (entropy) for SRAM power-on values [20]. The min-entropy of every bit ($bit_{i})$ in the SRAM power-on sequence can be calculated by Equations (1) and (2).

(1) $$H_{min}^{bit_{i}}=-log_{2}P_{i,max}$$

(2) $$P_{i,max}=\max\left(P_{i,1},P_{i,0}\right)$$

where $P_{i,1}$ ($P_{i,0}$) is the probability of the corresponding cell being initialized to ‘1′ (‘0′).

Assuming that every bit in the start-up sequence is independent, the average min-entropy of per bit can be defined as follows [19]:

(3) $$H_{min}^{bit}=\frac{\sum_{i=1}^{n}H_{min}^{bit_{i}}}{n}=\frac{-\sum_{i=1}^{n}log_{2}P_{i,max}}{n}$$

(4) $$0\leq H_{min}^{bit}\;\leq 1$$

where *n* represents the length (number of bits) of the start-up sequence. Unless otherwise specified, min-entropy hereinafter means$\;H_{min}^{bit}$.

Low min-entropy indicates that start-up values possess good repeatability and are suitable for extracting chip-specific fingerprints [19]. On the other hand, a higher min-entropy indicates that SRAM power-on values will be more random and can provide a great entropy source for the construction of a TRNG [21].

Thorough simulations and measurements of SRAM cells reveal that the mismatch of threshold voltage between P1 and P2 ($\Delta V_{th}$,$\left|\Delta V_{th}\right|=|V_{th,p1}-V_{th,p2}|$) dominates the power-on process [22,23,24]. Assuming $\left|\Delta V_{th}\right|$ is large enough (e.g., $\left|V_{th,p1}\left|\gg \right|V_{th,p2}\right|$), such a cell is called a strong cell. Strong cells have preferred (nonrandom) power-on values [21] and thus their occurrence must be minimized as much as possible for the TRNG construction in this paper.

On the other hand, there always exist some cells in which $\left|\Delta V_{th}\right|$ is very small ($\left|V_{th,p1}\right|\approx |V_{th,p2}|$); these are called noisy (weak) cells [24,25]. The power-on values of such cells are easy to flip between any two power-on measurements due to inevitable environmental noise. These noisy cells increase the entropy of the power-on sequence and provide a superb source of randomness, therefore making it possible to utilize SRAM as a TRNG [19].

## 2. Related Works and Our Contributions

As far as we know, studies on utilizing SRAM power-on values to construct TRNGs have newly risen in the last several years. Holcomb and Burleson et al. [15] first proposed the idea of extracting random numbers from SRAM power-on values. The main bottleneck of this type of application is that only a small percentage of SRAM bits behave with noisy power-on characteristics and the min-entropy generally does not exceed 0.1. Besides that, the data remanence effect [26] also severely limits the throughput of generated random bitstreams. To overcome the issue of randomness, several approaches have been recently proposed.

Aysu and Gulcan et al. [27] xored the raw SRAM power-on bytes multiple times to generate random numbers. In their implementation, 1024 bits of raw SRAM data is 8-fold XORed to obtain a 128-bit random string. Vincent and Erik et al. [19] proposed one method of conditioning an original power-on sequence into a true random seed using Secure Hash Algorithm 256 (SHA-256) and then instantiating a deterministic random bit generator (DRBG) with this seed to generate pseudorandom bitstreams. Due to low entropy, no less than 1600 bytes of SRAM power-on values must be condensed to generate a 256-bit true random seed in this work. In order to improve the randomness of the original SRAM power-on sequence, Kiamehr and Golanbari et al. [21] suggested leveraging the transistor aging effect to increase min-entropy to about 0.5. However, they did not carry out accelerated aging experiments to verify the scheme. In addition, the throughput of generated true random numbers was seldom taken into consideration in the previous works.

The performance of SRAM-based TRNG is strengthened specifically by the following main contributions in this work:

1. Ionization irradiation increases the min-entropy over 20 times, which is a remarkable randomness improvement upon the existing works. In other words, about only 5% of the SRAM cells are needed to generate the true random bitstreams of the same length as the ones before irradiation, which significantly promotes the utilization rate of memory cells and decreases the area and power consumption per random bit generation. Furthermore, putting SRAM chips that are powered off into the irradiation lab is the main operation in our randomness improvement scheme, and this has a much lower cost and is simpler to implement compared to designing a dedicated SRAM cell to enhance inherent noise sensitivity (as in [11]).
2. Throughput is another obstacle for utilizing SRAM as a TRNG because the power-down time between two power-on cycles must be long enough to avoid the degradation of the randomness of power-on values by the remaining charge. Many researchers even give up optimizing throughput [13,21,27,28]. Ionization irradiation can accelerate charge leakage and hence decrease the minimal power-down period between successive two power-on cycles from approximately 250 ms to about 1.5 ms, which makes a great contribution to the throughput of TRNG. It can generate National Institute of Standards and Technology (NIST) SP 800-22 compatible true random bitstreams with throughput of 178 Mbps, which is four times as high as that achieved in [19].

The rest of this paper is organized as follows: The entire TRNG design scheme is outlined in Section 3. The comprehensive improvements of total ionizing irradiation (TID) on SRAM power-on characteristics are described in detail in Section 4. Thereafter, the compact hardware postprocessing to achieve cryptographic quality randomness is implemented on FPGA in Section 5. Finally, conclusions are drawn in Section 6.

## 3. Proposed SRAM-Based TRNG Scheme

**Figure 2:**
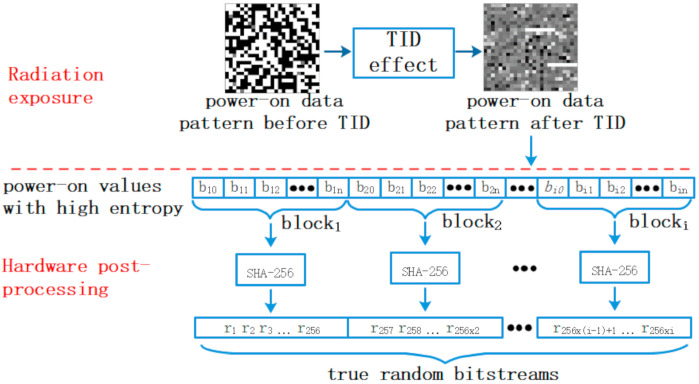
Flow of the proposed true random number generator (TRNG) scheme, including two stages: radiation exposure and hardware postprocessing.

The procedure of the proposed TRNG is shown in Figure 2. In the stage of radiation exposure, the randomness of SRAM power-on sequence is enhanced by TID. In the subsequent hardware postprocessing, SRAM is repeatedly powered on to get true random bitstreams continuously. During each power-on cycle, every n bits of power-on values are integrated into one block, which is conditioned into a 256-bit true random string with full entropy (i.e., the amount of entropy in the string is equal to its length [20,29]) by means of SHA-256 hash function.

In order for the output string of SHA-256 to have full entropy (256 bits), the amount of entropy at the input of the hash function should be at least 256 bits [19,30]. This transforms into the demand for the block size.

(5) $$H_{min}^{bit}\times n\geq 256$$

In addition, throughput of the true random bitstreams is also in the scope of this work.

(6) $$\begin{array}{c} throughput=\frac{amount\;of\;bits\;generated\;per\;cycle}{needed\;time}=\;\frac{S}{PD_{T}+SHA_{T}}=\frac{256\times i}{PD_{T}+i\times \frac{c}{f}}\\ =\frac{256\times \frac{m}{n}}{PD_{T}+\frac{m}{n}\times \frac{c}{f}}=\frac{256}{\frac{PD_{T}\times n}{m}+\frac{c}{f}} \end{array}$$

where S represents the amount of true random bits generated during per power-on cycle, $PD_{T}$ is the power-down period between two power-on cycles, $SHA_{T}$ is the time taken by SHA-256 calls, f represents the clock frequency of hardware platform used to perform SHA-256, c denotes the number of clock cycles taken to perform SHA-256 single call, i and n respectively represent the number of blocks and the size of single block and m expresses the capacity of SRAM memory.

It is obvious from Equation (6) that fast clock frequency (f) and large memory capacity (m) both are beneficial to throughput; short power-down period ($PD_{T}$) and small block size (n) are also preferred considering throughput.

However, the contradiction between throughput and randomness must be noticed. On the one hand, both block size and power-down time should be decreased as much as possible to improve throughput. However, power-down time must be long enough to avoid the remaining charge [26] between the two power-on cycles degrading the randomness of power-on values. Analogously, with too small of a block size, it be cannot guaranteed that the generated bitstreams will process full entropy [19,31].

In the next section, we will explain how to utilize TID to resolve the contradiction between randomness and throughput.

## 4. Impact of TID on SRAM Power-On Characteristics

In order to utilize the TID effect to improve SRAM power-on characteristics, there should be no supply voltage connected to the power pin of SRAM chip, and the other signal pins must also be grounded during radiation experiments, so the all transistors will undergo ionization irradiation with zero voltage bias. The detailed physical mechanisms behind this type of irradiation bias are illustrated in the Appendix A. The basic idea is described below.

Assume that there is a strong cell (e.g., $\left|V_{th,p1}\right|\gg |V_{th,p2}|$). In such case, the oxide field existing in P1 is weaker than that in P2 during irradiation with zero voltage bias. Although the electron–hole pairs generated per second are equal in both transistors, the amount of un-recombined holes that become trapped charges is lower in P1 due to a feebler oxide field. Therefore, the threshold voltage shift of P1 will be smaller than that of P2, and the threshold voltage of P2 will gradually catch up with P1 after multiple iterative irradiation experiments. In sum, the TID effect with zero voltage transforms a strong cell ($\left|V_{th,p1}\left|\ll \right|V_{th,p2}\right|$ or $\left|V_{th,p1}\left|\gg \right|V_{th,p2}\right|$) into a weak cell ($\left|V_{th,p1}\left|\approx \right|V_{th,p2}\right|$). As for a weak cell, it retains a noisy power-on characteristic under radiation, as the threshold voltages of both two P-channel Metal Oxide Semiconductor (PMOS) transistors shift almost equally.

**Figure 3:**
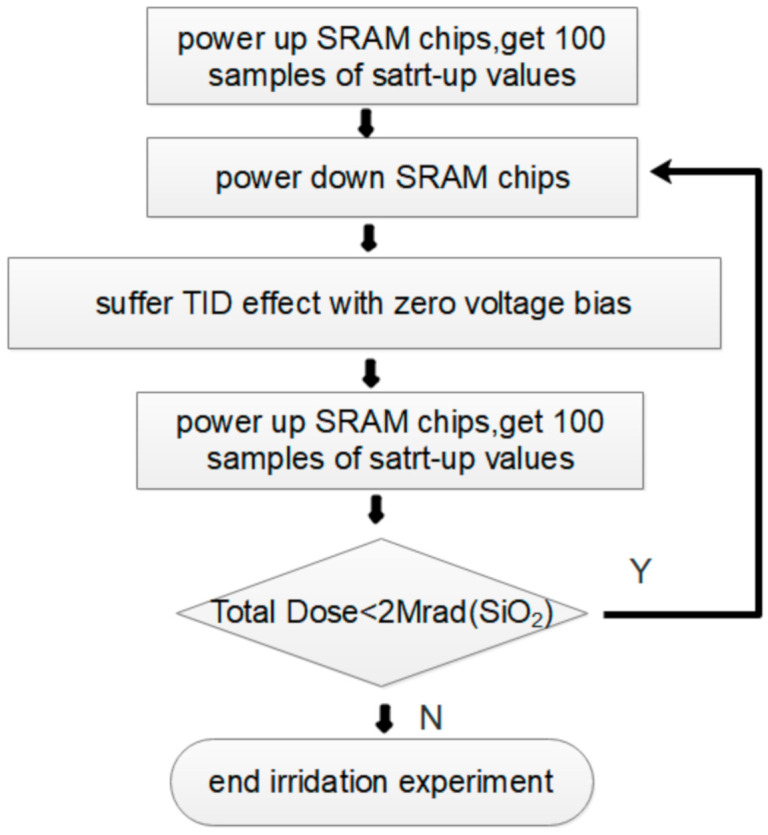
Steps of proposed SRAM power-on characteristics improvement utilizing irradiation exposure.

Considering the above factors, the randomness of all of the SRAM chip power-on values will be enhanced under irradiation with zero voltage bias. To assess the impact of TID on SRAM power-on characteristics quantificationally, irradiation experiments were performed on one type of commercial SRAM with a memory capacity of 1 Mbit, manufactured by 90 nm CMOS technology. Our experiments were carried out using the Cobalt-60 Irradiation Source of Peking University in China, and its dose rate is 50 Rad/s. Five SRAM chips (hereinafter denoted as Chip #1 to Chip #5) were randomly selected for testing. The experiment steps are shown in Figure 3.

Every tested chip was powered on, and its start-up values were sampled 100 times before irradiation, for contrast with the postirradiation cases. Thereafter, the chips were powered off and suffered ionization irradiation iteratively; for each additional 500 Krad(SiO_2_) doses, the chips were taken out to observe their power-on characteristics. To ensure that the normal read–write function of SRAM chips would not be damaged by the TID effect, 2 Mrad(SiO_2_) was chosen as the maximal irradiation dose.

**Figure 4:**
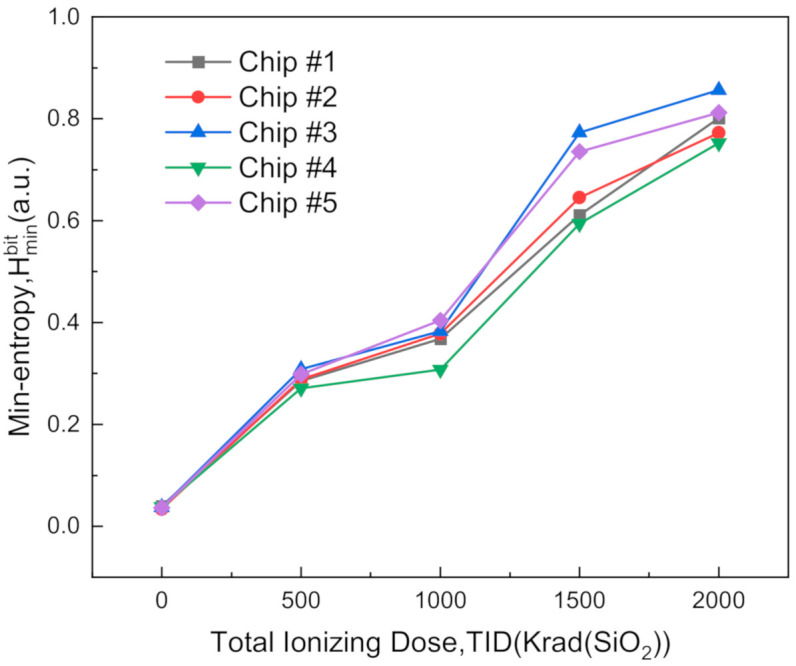
Min-entropy ($H_{min}^{bit}$) calculated at each dose step.

As an important indicator of randomness, the min-entropy ($H_{min}^{bit}$) was calculated at each dose step. As displayed in Figure 4, the min-entropy grew from about 0.03 to more than 0.75 as the total dose gradually increased to 2 Mrad(SiO_2_) for all tested chips, which exceeds a 20-fold increase. Besides the improvement of randomness, the rising min-entropy can also help to decrease the block size (n) and thus increase the throughput in the subsequent hardware postprocessing, as discussed in Section 5.

It can be also observed that the sample chip with the worst randomness improvement was the Chip #4; this chip is regarded as one special sample for evaluating the proposed TRNG scheme conservatively in the following statements.

**Figure 5:**
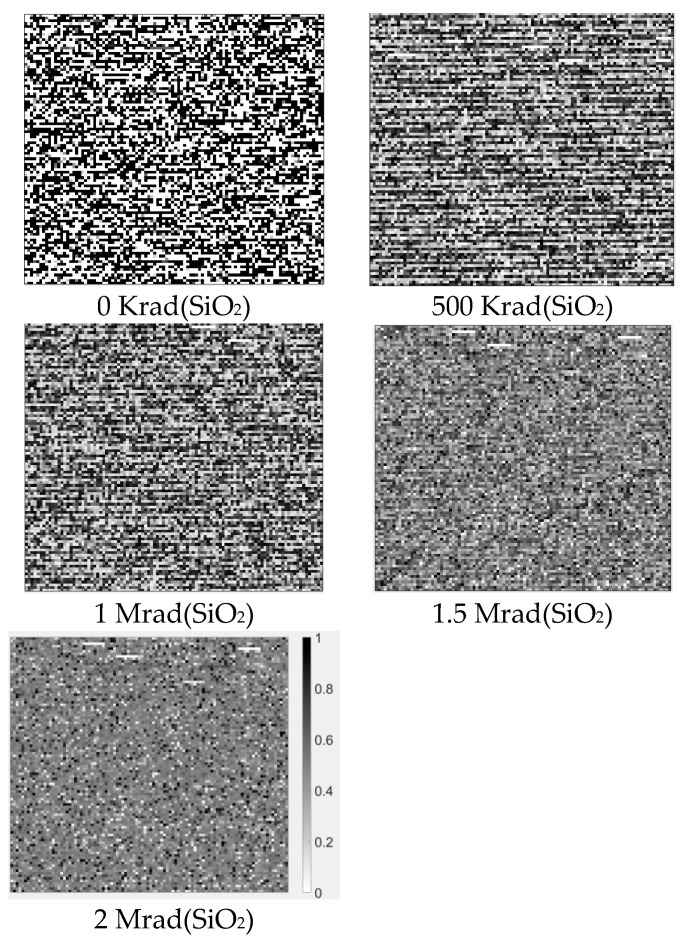
P0 of SRAM cells in Chip #4 under different doses. The darker the grid, the higher probability of the corresponding cell being initialized to ‘0′.

Figure 5 shows the probabilities of cells in Chip #4 being initialized to ‘0′ (P0). Before irradiation, the P0 value of most cells was either 0 or 1, which means that the SRAM power-on values are not random. However, by applying irradiation iteratively, P0 of most cells gradually converged close to 0.5. This reveals the obvious randomness improvement of the SRAM power-on values.

In addition to the assessment of randomness, the data remanence effect, which exerts strong influence on the throughput of the proposed TRNG, was characterized. One SRAM chip was powered down after initializing its cells to all 0′s or all 1′s; following a short period, the chip was repowered on. If the power-down period ($PD_{T}$) was too short, then the data would deterministically revert to the previous written state. As the power-down period increased, the charges remaining in the parasitic capacitors would gradually be reduced so that more and more SRAM cells would turn over their power-on values which were opposite to the previous written ones [26]. If the power-down period was long enough, then the charges would leak completely and the SRAM chip would power up to its random state (i.e., the data stored in about 50% of cells would flip). We denote the shortest power-down period making the portion of flipped cells converge to a stable value near 50% as $PD_{T\_min}$. When SRAM power-on values are utilized to construct a TRNG, the power-down period between any two power-on cycles must not be lower than $PD_{T\_min}$ to avoid the influence of data remanence and guarantee randomness of the generated bitstreams.

**Figure 6:**
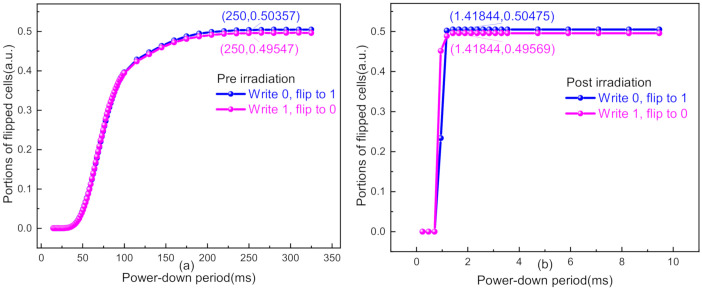
Portions of flipped cells at different power-down periods (**a**) before irradiation and (**b**) under 2 Mrad(SiO_2_).

Figure 6a shows the portions of flipped cells in Chip #4 when the power-down period was swept from 10 to 325 ms before irradiation. In both write ‘1′ and write ‘0′ cases, $PD_{T\_min}$ reached up to about 250 ms. Figure 6b displays the sweep results of when Chip #4 was exposed to irradiation of 2 Mrad(SiO_2_), and $PD_{T\_min}$ took only approximately 1.4 ms for both write ‘1′ and write ‘0′ cases. The dramatic decrease of $PD_{T\_min}$ will make a great contribution to the throughput of a TRNG.

## 5. Hardware Postprocessing

As depicted in Section 4, irradiation exposure can make SRAM chips a great entropy source. The decrease of $PD_{T\_min}$ also makes it possible to generate random bitstreams with high throughput. However, it is still necessary to rigorously judge the viability of utilizing the raw power-on values as true random bitstreams. For this purpose, the National Institute of Standards and Technology (NIST) SP 800-22 test was carried out on the power-on sequence of Chip #4 for both pre- and postirradiation cases. As is shown in Table 1, the power-on sequence before irradiation failed almost all of the tests due to low entropy and strong data remanence effect at the power-down period of 3 ms (as is illustrated in Figure 6a); the power-on sequence under 2 Mrad(SiO_2_) dose showed a great randomness improvement in behavior but still could not pass all the tests, needing postprocessing to improve the statistical properties [32].

Actually, the raw random bits of many TRNGs become biased and cannot meet the NIST SP800-22 test standards [10]; therefore, some postprocessing methods are commonly employed on the raw random bits to generate the true random numbers with full entropy that can pass all NIST test items. The postprocessing schemes include but are not limited to digital postprocessing using the XOR function [33,34,35,36,37], Von Neumann extractor [38,39,40,41], Hash functions (e.g., Secure Hash Algorithm 256, (SHA-256)) [11,19,42] and even circuit calibration technologies [10,43]. SHA-256 was selected to complete conditioning in this paper as it exists in many encrypted communication coprocessors [42] and its use might not increase overheads in practical applications.

We performed SHA-256 on Xilinx XC7Z020 [44] with clock frequency of 200 MHz, and our implementation could perform one hash at 64 clocks. By performing this kind of compact postprocessing on the power-on values under 2 Mrad(SiO_2_), conditioned random bitstreams with throughput of 178 Mbps were generated, which were able to pass all NIST test items, as shown in Table 1. For the sake of contrast, the same operations were implemented on the power-on values before irradiation, which could generate bitstreams with the same throughput as the ones under 2 Mrad(SiO_2_). However, the conditioned bitstreams still could not pass all tests, as displayed in Table 1.

It can be summarized from the above NIST test results that high throughput requires small block size (i.e., input string length of SHA-256) and short power-down period. The power-on sequences under 2 Mrad(SiO_2_) could completely meet these two requirements and hence generate true random bitstreams with throughput up to 178 Mbps. However, power-on sequences without irradiation collected at such a small block size (370 bits) and short power-down period (3 ms) behave too poorly in terms of randomness due to large mismatches in SRAM cells and strong data remanence effect.

Additionally, it is necessary to further investigate the upper limit of throughput on the promise of generating true random numbers with full entropy by means of the proposed hardware implementation. As is revealed by Equation (6), the throughput is mainly constrained by block size (n), power-down period ($PD_{T}$), memory capacity (m) and clock frequency (f). Under the promise of the conditioned bitstreams behaving with full entropy, the lower bound of block size can be defined by Equation (5) (i.e., $n\geq \frac{256}{H_{min}^{bit}}$), and $PD_{T}$ cannot be shorter than $PD_{T\_min}$ to eliminate the impact of remaining charge. Thus, the upper bound of throughput can be approximated as follows:

(7) $$throughput\;=\frac{256}{\frac{PD_{T}\times n}{m}+\frac{64}{f}}\leq \;\frac{256}{\frac{PD_{T}\times 256}{m\times \;H_{min}^{bit}}+\frac{64}{f}}\;\leq \;\frac{\;256}{\frac{PD_{T\_min}\times 256}{m\times \;H_{min}^{bit}}+\frac{64}{f}}$$

$PD_{T\_min}$ and $H_{min}^{bit}\;$of Chip #4 were measured in Section 4 for both pre- and postirradiation situations and thus can be seen as constants here. Next, the maximum throughputs at different memory capacities and clock frequencies are compared for both pre- and postirradiation situations.

**Figure 7:**
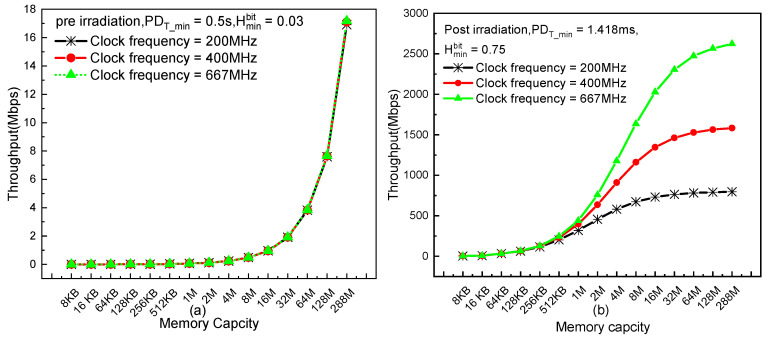
Maximum throughput at different memory capacities and clock frequencies: (**a**) preirradiation and (**b**) under 2 Mrad(SiO_2_).

Before irradiation, the low entropy and long power-down period heavily encumber throughput. As displayed in Figure 7a, the three almost coincident curves indicate that neither large memory capacity nor high clock frequency can produce reasonable throughput, as it cannot reach 1 Mbps even with memory capacity of 8 Mb and clock frequency of 667 MHz. Thus far, we can conclude that it is unpractical to generate true random bitstreams with high speed by utilizing the raw power-on values without irradiation.

For the chip under 2 Mrad, random bitstreams with high throughput can be obtained due to the mutual improvements of both randomness and throughput benefiting from irradiation exposure. As shown in Figure 7b, both large memory capacity and high clock frequency vigorously motivate the throughput. It also shows that the throughput can be widely adjusted according to the application requirements by using different clock frequencies and memory capacities. Of course, at a certain clock frequency, the throughput gradually reaches its saturation point with the memory capacity increase. This characteristic can also provide a reference for optimized design of a TRNG.

Finally, a comparison with prior works is presented in Table 2. The entropy of SRAM power-on sequence obtains more significant improvement by impact of ionizing irradiation, and our proposed SRAM-based TRNG can generate true random bitstreams with higher throughput, rather than obtaining a true random seed to instantiate a deterministic random bit generator (DRBG) and generate pseudorandom bitstreams [21].

## 6. Conclusions

In our work, a novel SRAM-based TRNG is proposed; its implementation consists of two phases: irradiation exposure with zero voltage bias and hardware postprocessing. During the irradiation phase, the TID effect is utilized to make SRAM cells less mismatched and hence to obtain more random power-on values. The experiment results show that the min-entropy of SRAM power-on values can increase over 20 times as ionization dose is accumulated to 2 Mrad(SiO_2_). Besides, the minimal power-down period between two successive power-on cycles can decrease from approximately 250 ms to about 1.5 ms, which makes a great contribution to the throughput of a TRNG. In the subsequent hardware postprocessing, SRAM chip should be repeatedly powered up; during each power-on cycle, SRAM power-on values are conditioned into true random bitstreams by means of SHA-256. One implementation on FPGA can generate bitstreams with throughput of 178 Mbps, which can pass all NIST SP 800-22 tests.

## Figures and Tables

**Table 1:** National Institute of Standards and Technology (NIST) test results for different bitstreams. The list of applied tests is as follows: (1) monobit frequency test, (2) block frequency test, (3) run test, (4) longest run, (5) binary matrix rank, (6) spectral test, (7) non-overlapping template matching, (8) overlapping template matching, (9) Maurer’s universal statistic test, (10) linear complexity test, (11) serial test, (12) approximate entropy test, (13) cumulative sums (forward) test and (14) cumulative sums (reverse) test.

	Pre-Irradiation (Power-Down Period ($PD_{T}$) = 3 ms, Block Size (n) = 370)		Under 2 Mrad(SiO_2_) (Power-Down Period ($PD_{T}$) = 3 ms, Block Size (n) = 370)	
	Power-on Sequence	Conditioned Bitstreams	Power-on Sequence	Conditioned Bitstreams
1	✕	✓	✓	✓
2	✕	✓	✓	✓
3	✕	✓	✓	✓
4	✕	✓	✓	✓
5	✓	✕	✓	✓
6	✕	✕	✓	✓
7	✕	✓	✓	✓
8	✕	✓	✓	✓
9	✕	✓	✕	✓
10	✓	✓	✓	✓
11	✕	✕	✓	✓
12	✕	✕	✕	✓
13	✕	✓	✓	✓
14	✕	✓	✓	✓

**Table 2:** Comparison with prior works.

	[1]	[21]	[11]	[45]	This Work
Min-entropy of SRAM power-on values	0.02	0.5	0.89	0.103	0.75
Type of conditioned bitstreams	Pseudorandom	--	True random	Pseudorandom	True random
Throughput of conditioned bitstreams	40 Mbps	--	--	--	178 Mbps
NIST SP 800-22 tests on conditioned bitstreams	Pass	--	Pass	Pass	Pass
Postprocessing	Hash	--	Hash	Hash	Hash
Normal storage function	Yes	Yes	No	Yes	Yes

**Figure A1:**
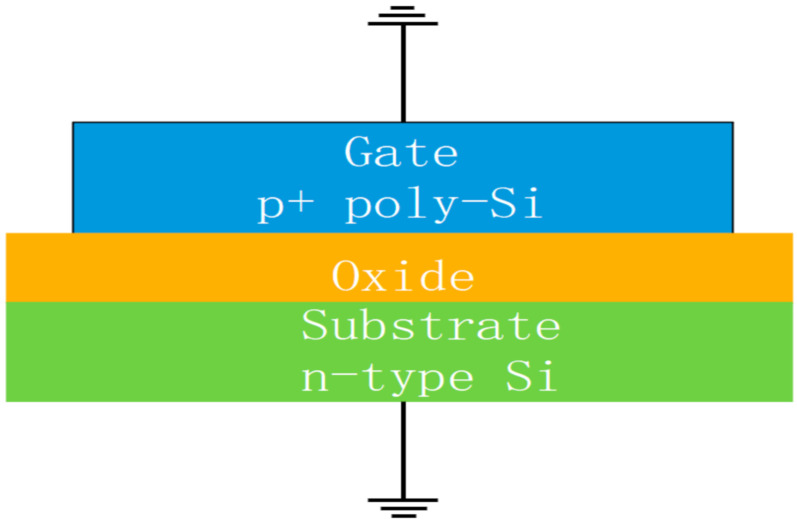
Schematic cross section of a P-channel MOSFET (PMOS) capacitor with zero voltage bias.

**Figure A2:**
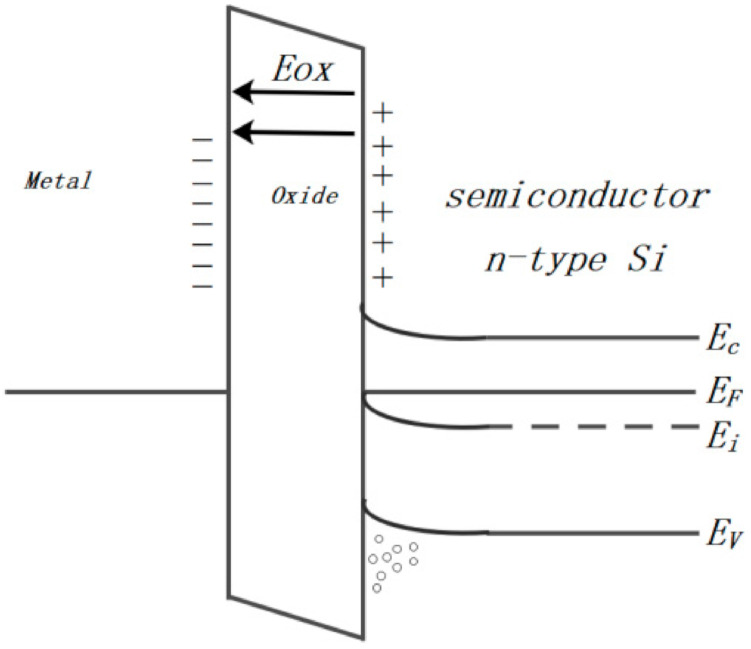
The energy band becomes balanced and built-in field arises due to charge motion.

## Appendix A

A P-channel MOSFET (PMOS) with zero voltage bias can be simplified into an MOS capacitor for analysis, as shown in Figure A1. It consists of a conducting gate electrode (here, heavily doped p+ polysilicon) on top of a thin layer of silicon dioxide grown on a silicon substrate [46].

The work function (Φ) [46] is the energy required to extract an electron from the Fermi energy ($E_{F})$ to the vacuum level. Φ is determined by the property of material; the work function of p+ poly-Si ($\Phi_{m}$) is approximately 5.27 eV, and the work function of n-type Si ($\Phi_{s}$) is approximately (4.15 eV+0.56 eV$-\left|\Phi_{F}\right|$) [46,47].

(A1) $$\Phi_{m}>\Phi_{S}$$

So, the fermi energy of p+ poly-Si$\;(E_{FM})$ is lower than that of n-type Si$\;(E_{Fs})$.

(A2) $$E_{FM}<E_{Fs}$$

It is obvious that the energy band is not balanced when the three components are piled up to formulate the MOS structure shown in Figure A1, so the charge motion will be developed to flatten energy band as displayed in Figure A2. The electrons are attracted to the gate electrode and holes are collected at the interface of n-type Si. As the Fermi levels line up, electric field is developed in both the oxide and silicon [46].

The voltage drop across oxide is the flat band voltage ($V_{FB})$ of MOS.

(A3) $$|V_{ox}|=\;(\Phi_{m}-{\;\Phi }_{S})/q={\;\Phi }_{ms}/q$$

The oxide field is

(A4) $$E_{ox}=\frac{\left|V_{ox}\right|}{t_{ox}}=\frac{\Phi_{ms}}{qt_{ox}}$$

where $t_{ox}$ is the thickness of oxide.

The threshold voltage of PMOS ($V_{th})$ can be expressed as follows:

(A5) $$V_{th}=-\frac{\sqrt{4\varepsilon_{s}qN_{a}\psi_{B}}}{C_{ox}}-2\psi_{B}+\frac{\phi_{ms}}{q}$$

(A6) $$|V_{th}|=\frac{\sqrt{4\varepsilon_{s}qN_{a}\psi_{B}}}{C_{ox}}+2\psi_{B}-\frac{\phi_{ms}}{q}$$

The substrate doping concentration ($N_{a}$) and thickness of oxide ($t_{ox}$) will be slightly different between transistors due to process variations that cannot be fully controlled [48,49]. These differences are actually the main sources of the mismatch of threshold voltage$\;\left(\Delta V_{th}\right)$.

Assume there is significant mismatch between the threshold voltage of twoPMOS; i.e.,

(A7) $$\left|V_{th,p1}\right|>|V_{th,p2}|$$

If the mismatch is mainly contributed by the difference in the thickness of oxide and there is almost no difference between substrate doping concentrations, then

(A8) $$\;\phi_{ms1}\;=\;\phi_{ms2}$$

The unit-area capacitance ($C_{ox}$) of P1 should be smaller than that of P2 according to Equation (A6).

(A9) $$C_{ox1}=\frac{\varepsilon_{ox}}{t_{ox1}}<C_{ox2}=\frac{\varepsilon_{ox}}{t_{ox2}}$$

Thus, the oxide in P1 should be thicker.

(A10) $$t_{ox1}>t_{ox2}$$

It can be concluded that the oxide field of P1 is weaker.

(A11) $$E_{ox1}=\frac{\phi_{ms1}}{qt_{ox1}}<E_{ox2}=\frac{\phi_{ms2}}{qt_{ox2}}$$

If the mismatch is mainly deduced by the difference between substrate doping concentrations ($N_{a}$), then the substrate of P1 should be doped more heavily than that of P2 according to Equations (A6) and (A7).

(A12) $$N_{a1}>N_{a2}$$

For n-type-Si, the increase of doping concentration will motivate the energy band of the substrate to rise up.

(A13) $$\phi_{ms1}\;<\;\phi_{ms2}$$

This phenomenon will make the oxide field of P1 weaker.

(A14) $$E_{ox1}=\frac{\phi_{ms1}}{t_{ox1}}<E_{ox2}=\frac{\phi_{ms2}}{t_{ox2}}$$

To conclude, whether substrate doping concentration or thickness of oxide contributes to the mismatch of $V_{th}$, there always exists a smaller oxide field in the PMOSFET possessing higher $V_{th}$.

In what follows we will probe into how the threshold voltages of P1 and P2 shift under TID effect with zero voltage bias. When an MOS device is exposed to ionizing radiation, electron–hole pairs are formed uniformly throughout the oxide [50]. The charge pair volume density per rad is generally estimated to be $G_{0}$ = 8.1 × 10^12^ cm^–3^/rad [51], but this initial density is quickly reduced by the initial recombination process.

After escaping recombination, the rate at which holes are injected into the oxide by radiation is given by [50] as

(A15) $$\frac{dN}{dt}=G_{0}RY$$

where $G_{0}\;$= 8.1 × 10^12^ cm^–3^/rad in SiO_2_, R is the dose rate and Y is the yield of electron–hole pairs (un-recombined). $G_{0}$ and R are both constant for devices in actual experiments, but charge yield (Y) varies because only a fraction of the holes formed will escape initial recombination.

Y is dependent on the electric field across oxide ($E_{ox})$; an empirical formula estimating Y with $E_{ox}$ is written as follows [50]:

(A16) $$Y≅0.49[1+\tanh\left(1.2log_{10}\left(E_{ox}\right)\right)]$$

For a strong cell (e.g., $\left|V_{th,p1}|>|V_{th,p2}\right|$,$\Delta V_{th}=|V_{th,p1}\left|-|V_{th,p2}\right|$), no matter whether $\Delta V_{th}$ is induced by the variations of substrate or oxide, the built-in field existing in P1 will be lower than that in P2 according to Equations (A11) and (A14).

(A17) $$E_{ox1}<E_{ox2}$$

When undergoing total ionizing dose radiation, electron–hole pairs generated per second are equal in both transistors.

(A18) $$G_{01}R=G_{02}R$$

However there are less un-recombined electron-hole pairs in P1 according to Equation (A16).

(A19) $$Y_{1}<Y_{2}$$

Then, P1 will process less holes, which finally become positive trapped charges and contribute to the negative $V_{th}\;$shift.

(A20) $$\Delta N_{1}<\Delta N_{2}$$

Thus, the $V_{th}\;$shift of P1 will be smaller, and the threshold voltage of P2 will gradually catch up with P1 after multiple iterative irradiation experiments.

(A21) $$\left|V_{th,p1}\right|\approx |V_{th,p2}|,\;\Delta V_{th}\approx 0$$
